# Modulation of lncRNAs and oxidative stress related genes by N-acetylcysteine and S-methylcysteine in rotenone-induced Parkinson's disease

**DOI:** 10.1016/j.bbrep.2025.102328

**Published:** 2025-10-25

**Authors:** Sahar Yaqubi, Bagher Seyedalipour, Mohammad Karimian

**Affiliations:** Department of Molecular and Cell Biology, Faculty of Basic Sciences, University of Mazandaran, Babolsar, Iran

**Keywords:** Parkinson's disease, Oxidative stress, N-Acetyl cysteine, S-Methyl cysteine, Nrf2/Ho-1 pathway

## Abstract

Oxidative stress and changes in lncRNA expression are key factors in the pathophysiology of Parkinson's disease. This study investigated the protective effects of N-acetylcysteine and S-methylcysteine on the expression of long non-coding RNAs and oxidative stress-related genes in the brain, as well as the activity of antioxidant enzymes in the brain and serum of mice with rotenone-induced Parkinson's disease. In this experimental study, 56 male BALB/c mice were utilized and treated continuously for 10 days. Gene expression of superoxide dismutase, glutathione peroxidase, catalase, *Nrf2*, *Ho-1*, and long non-coding RNAs *Malat1*, *Neat1*, and *Gas5* in the brain was analyzed using real-time PCR. Biochemical assays measured antioxidant enzyme activities, malondialdehyde levels, and total antioxidant capacity in brain tissue and serum. A bioinformatics approach, including molecular docking and the construction of a gene interaction network, was also performed. Our data showed decreased expression of antioxidant genes and *Nrf2* and *Ho-1* regulatory factors in the Parkinson's group, which were significantly restored by N-acetylcysteine and S-methylcysteine treatments. Long non-coding RNAs were elevated in the Parkinson's disease model and reduced by interventions. Antioxidant enzyme activity and oxidative stress markers were significantly improved by N-acetylcysteine, S-methylcysteine, and their combination. Molecular docking suggested stable interactions of these compounds with antioxidant enzymes. The interaction network highlights *Nrf2* as a central regulator of antioxidant genes, modulated by specific lncRNAs. Findings support the neuroprotective role of N-acetylcysteine, S-methylcysteine through activation of the *Nrf2*/*Ho-1* pathway, modulation of long non-coding RNAs, and oxidative stress improvement in Parkinson's disease.

## Highlights

• NAC and SMC regulated the expression of lncRNAs and antioxidant genes in a Parkinson's model.
• Antioxidant enzyme activities and total antioxidant capacity were improved by NAC and SMC.
• The Nrf2/Ho-1 pathway, as a central regulator of oxidative stress, was activated by NAC and SMC.

## Graphical abstract

**Figure:**
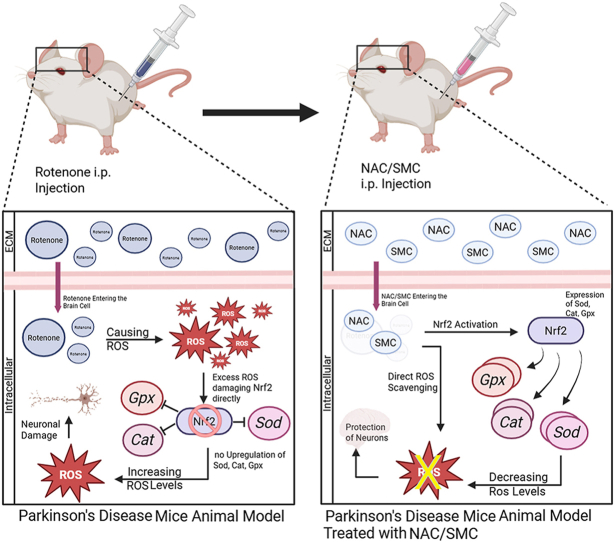

## 1 Introduction

Parkinson's disease (PD) is one of the most common neurodegenerative diseases associated with progressive damage to dopaminergic neurons in the substantia nigra of the brain [1]. PD leads to motor symptoms such as tremor, muscle rigidity, and bradykinesia, and in more advanced stages, cognitive and psychiatric problems are observed [2]. The disease occurs in people over the age of 60. However, both genetic predispositions and environmental influences may elevate the risk even at younger ages. Various studies suggest that oxidative stress-induced neurodegeneration, neuronal inflammation, and abnormal aggregation in dopaminergic neurons are among the primary mechanisms involved in the disease's pathophysiology [3].

One important pathway involved in the development of PD is oxidative stress in neurons, which leads to decreased mitochondrial function, cell damage, and ultimately neural death [4]. Oxidative stress occurs when the combination of various reactive oxygen species (ROS) and antioxidant defense mechanisms is disrupted. In patients with PD, abnormal accumulation of ROS and free radicals leads to the lipids, proteins, and DNA oxidation and damages vital cellular structures. This process ultimately leads to apoptotic and necrotic pathway activation and reduction of dopaminergic neuron function, resulting in the generation of motor effects in the substantia nigra [5]. In response to this disease stress, several defense mechanisms are controlled through molecular antioxidant pathways. One of these pathways is related to nuclear factor erythroid 2-related factor 2 (Nrf2), which is a key regulator of antioxidant defense [6]. Nrf2 is normally sequestered in the cytoplasm by the Keap1 promoter complex, but upon exposure to oxidative stress, it dissociates from this complex and translocates to it, where it binds to antioxidant response elements (AREs) to increase the expression of target genes [7]. Among the most important genes regulated by Nrf2 are hemeoxygenase-1 (*Ho-1*), superoxide dismutase (*Sod*), glutathione peroxidase (*Gpx*), and catalase (*Cat*), which are involved in the repair of ROS-induced damage [8]. Recent studies have shown that in patients with PD, this pathway is not functioning properly and is not properly regulated, leading to antioxidant protection and increased neuronal damage [9].

In recent years, long non-coding RNAs (lncRNAs) have been recognized as key regulators in the pathogenesis of PD [10]. Certain lncRNAs influence oxidative stress responses by modulating the Nrf2 signaling pathway and its downstream targets. Among them, Malat1, Neat1, and Gas5 have received particular attention due to their roles in redox homeostasis and neurodegeneration. It has been reported that *Malat1* suppresses *Nrf2* through epigenetic mechanisms, leading to increased inflammasome activation and elevated ROS generation in both Parkinson's disease mouse models and microglial cells [11]. Gas5, a stress-responsive lncRNA, has been associated with impaired antioxidant capacity and enhanced neuronal vulnerability under oxidative conditions [12,13]. It was found that the upregulation of *Neat1* led to the suppression of the Nrf2 signaling pathway, which was subsequently associated with neuronal damage [14]. Regarding these findings, investigating the altered expression of Malat1, Neat1, and Gas5 may provide novel insights into lncRNA-mediated regulation of the Nrf2/Ho-1 pathway in PD.

Given the key role of oxidative stress in the pathophysiology of PD, antioxidant compounds have been used as a potential therapeutic strategy to reduce neuronal damage. Among them, two compounds, N-Acetyl Cysteine (NAC) and S-Methyl Cysteine (SMC), have been proposed as emerging pharmacological agents due to their ability to regulate antioxidant pathways and reduce ROS-induced damage. NAC is an acetylated derivative of the amino acid cysteine, which is known as a precursor of glutathione (GSH), one of the most important intracellular antioxidants [15]. This compound provides significant protection to neurons by regulating the Nrf2 pathway, increasing the expression of antioxidant genes, and improving mitochondrial function. In addition, NAC has also been shown to have anti-inflammatory properties and can reduce the damage caused by the neurodegenerative brain by reducing the expression of pro-inflammatory cytokines [16]. On the other hand, SMC, which is found in food sources such as garlic and onions, has potent antioxidant and anti-inflammatory properties. SMC can increase the activity of antioxidant enzymes and protect cell structures through its ability to produce ROS, safeguarding dopaminergic neurons from damage caused by oxidative stress. Studies show that SMC can activate Nrf2 and increase the expression of antioxidant genes related to it [17]. These unique properties make NAC and SMC considered as two agents in reducing neuronal damage and improving cellular function in neurodegenerative diseases, including PD. expression in an animal model of rotenone-induced PD.

Previous research has established the role of oxidative stress in the pathogenesis of PD, particularly in models induced by rotenone. However, there is a lack of studies examining the combined protective effects of NAC and SMC on oxidative damage in this context. Furthermore, few studies have investigated how these compounds influence the expression of antioxidant-related genes and enzyme activity. Therefore, this study aimed to investigate the protective effects of NAC and SMC on oxidative stress and the regulation of antioxidant gene expression and enzyme activity in an animal model of rotenone-induced PD.

## 2 Materials and methods

### 2.1 Chemicals

The materials used in this study included NAC (Sigma-Aldrich, St. Louis, MO, USA), SMC (Sigma-Aldrich, St. Louis, MO, USA), Rotenone (Sigma-Aldrich, St. Louis, MO, USA), Levodopa (Sigma-Aldrich, St. Louis, MO, USA), Ketamine (Sigma-Aldrich, St. Louis, MO, USA), Xylazine (Sigma-Aldrich, St. Louis, MO, USA), Gpx assay kit (NagpixTM Kit, Navand Salamat Co., Urmia, Iran), RNA extraction kit (Pars-Tous, Mashhad, Iran), cDNA synthesis kit (Pars-Tous, Mashhad, Iran), PCR related items (Pishgam, Tehran, Iran), SYBR Green 2X Mix (SinaClon, Tehran, Iran), and primers used in PCR (Pishgam, Tehran, Iran).

### 2.2 Laboratory Animals and experimental groups

**Fig. 1:**
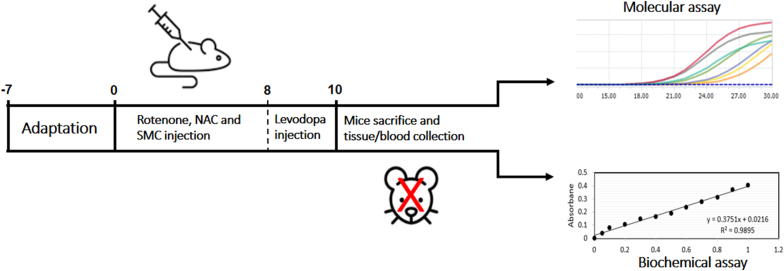
Experimental flow chart of the study. The diagram summarizes the overall experimental design, including treatment administration, sample collection, and biochemical assessments, as well as molecular analyses.

In this study, 56 adult male mice weighing 20–25 g were obtained from the Pasteur Institute, Amol, Iran. The animals were kept in five separate cages at a temperature of 22 ± 2 °C, under a photoperiod of 12 h' light and 12 h' dark, and at a humidity of 65 % ± 5 %. They were fed ad libitum with water and standard rodent chow. Also, before the start of the experiments, the animals were kept for at least one week to adapt to the laboratory conditions. The mice were randomly divided into seven groups; Control group (Con) received no substance, Sham group (Sham) only received DMSO solvent, PD group (PD) received rotenone at a dose of 2.75 mg/kg/day, PD group receiving NAC (PD + NAC) received rotenone at a dose of 2.75 mg/kg/day and NAC at a dose of 80 mg/kg/day, PD group receiving SMC (PD + SMC) received rotenone at a dose of 2.75 mg/kg/day and SMC at a dose of 100 mg/kg/day, PD group receiving NAC + SMC (PD + NAC + SMC) received rotenone at a dose of 2.75 mg/kg/day, NAC at a dose of 80 mg/kg/day and SMC at a dose of 100 mg/kg/day, and positive control group (PC): received rotenone at a dose of 2.75 mg/kg/day and then Levodopa at a dose of 8 mg/kg/day. PD was induced by intraperitoneal injection of rotenone at a dose of 2.75 mg/kg/day for 10 days. This method was chosen as a standard model for inducing PD-like lesions in mice based on a study conducted by Cannon et al. in 2009 [18]. NAC at a dose of 80 mg/kg/day and SMC at a dose of 100 mg/kg/day were used according to previous scientific literature [15,19]. These compounds were administered intraperitoneally for 10 days, individually and in combination, to different groups. As a positive control, Levodopa was administered at a dose of 8 mg/kg/day for 2 days in a separate group to compare the effects of the study treatments with the approved treatment [20]. The selection of toxicants/compounds and their doses was guided by prior reports validating their efficacy, stability, and safety for in vivo use under comparable experimental conditions. However, after the treatment procedure, mice were anesthetized with 100 mg/kg and 10 mg/kg of ketamine and xylazine, respectively. The brain was dissected and stored at −80 °C for subsequent enzymatic and gene expression analyses. In addition, serum was separated from the mice's blood and preserved at −80 °C for biochemical analyses. The experimental flow chart of the study is presented in Fig. 1 to illustrate the overall design and workflow of the research. All steps of this study were performed in accordance with the care guidelines outlined in the National Institutes of Health Guide for the Care and Use of Laboratory Animals (NIH PUBLICATIONS NO. 8023, REVISED 1978). The care and use of animals was approved by the Ethics Committee of the University of Mazandaran, Babolsar (Code: IR.UMZ.REC.1403.092).

### 2.3 Examination of gene expression using real-time PCR

Real-Time PCR was used to evaluate the expression changes of the studied genes. Total RNA was extracted from the brains of all mice were processed using a commercial kit, following the manufacturer's instructions. The quantity and quality of the extracted RNA were assessed using 1 % agarose gel electrophoresis and converted to cDNA using another commercial kit. Real-Time PCR reaction was performed in a final volume of 20 μl containing 10 μl SYBR Green Master Mix (2x), 0.5 μl each of the forward and reverse primers (initial concentration: 10 pmol/μl), and 30 ng of diluted cDNA. *Gapdh* was used as an internal control in this process. Real-Time PCR reaction was performed using the Roche LightCycler 96 Real-Time PCR system (Roche, Germany). The sequences of the primers designed using Primer 3 software and their annealing temperatures are given in Table 1. Data were normalized based on the expression level of the *Gapdh* gene and relative changes in expression were calculated using the 2^−ΔΔCt^ method. The accuracy of the Real-Time PCR reaction was confirmed by melting curve analysis.

**Table 1:** Characteristics of specific primers.

Gene Name	Accession Number	Primer Sequence (3’→5′)	Primer annealing temperature (°C)	PCR Product Size
***Cat***	NM_009804.2	F: CGCAATCCTACACCATGTCG	57	226-bp
		R: TCAGGAATCCGCTCTCTGTC		
***Gpx***	NM_001329527.1	F: TGACCGACCCCAAGTACATC	59	217-bp
		R: CAGCCAGTAATCACCAAGCC		
***Sod***	NM_011434.2	F: AGAGAGGCATGTTGGAGACC	59	159-bp
		R: CCACCTTTGCCCAAGTCATC		
***Nrf2***	NM_010902.5	F: ACTTTGTGTCAGCTGCAGTG	59	204-bp
		R: TGGAAGTCACTGCCCTAAGG		
***Ho-1***	NM_010442.2	F: AAACAGGAAAGTGGTGGCAC	57	232-bp
		R: GCTTGCTTGAGATCGACCAG		
***Malat1***	NR_002847.4	F: GTCAAACAGGGCAAGATGGG	59	204-bp
		R: CCTCTGGAGTCCGATACCTG		
***Neat1***	NR_131049.1	F: TGCTTTGTTTGCCTGACTCC	57	232-bp
		R: TGAAACTACCAGGCTGAGGG		
***Gas5***	NR_003633.3	F: GAATGTTCACCTGGACCTGC	57	219-bp
		R: ACAGGAGTGGAGGTAAAGGC		
***Gapdh***	NM_001411843.1	F: CGACTTCAACAGCAACTCCC	59	217-bp
		R: GAGTTGGGATAGGGCCTCTC		

### 2.4 Antioxidant enzyme activity and oxidative stress indices

To assess the oxidative status, antioxidant enzyme activity and oxidative stress indices were analyzed in serum and brain tissue homogenates. Catalase enzyme activity was determined by the Aebi method [21]. In this method, by definition, one Cat unit is the amount of enzyme that is capable of degrading 1 μmol of H_2_O_2_ in 1 min at 25 °C. In this experiment, the decrease in absorbance of the solution at a wavelength of 240 nm was measured over 3 min and at 30-s intervals. The activity of the Gpx enzyme was examined using the NagpixTM kit (Navand Salamat Co., Urmia, Iran). This enzyme plays a key role in the reduction of lipid peroxides and the conversion of H_2_O_2_ to water. The Gpx reaction involves the oxidation of reduced glutathione (GSH) to oxidized glutathione (GSSG) and then the reduction of GSSG to GSH by glutathione reductase in the presence of NADPH. Gpx activity was assessed by measuring the consumption of NADPH, with optical absorbance recorded at 340 nm. The activity of the superoxide dismutase enzyme was examined by the Marklund & Marklund method [22]. In this method, the inhibition of superoxide anion production in the presence of Sod was determined. The absorbance of the samples was measured at a wavelength of 560 nm, and the data were analyzed based on a standard curve. Total antioxidant capacity (TAC) levels were measured using the Ferric Reducing Ability of Plasma (FRAP) method [23]. In this method, 50 μL of sample was mixed with 1.5 mL of FRAP reagent, and after 10 min incubation at 37 °C, the absorbance was read at 593 nm. TAC levels were calculated based on the Fe^2+^ standard curve, and the results were reported in micromolar for serum and micrograms per gram of brain tissue weight. To assess lipid peroxidation, malondialdehyde (MDA) levels were determined using the Thiobarbituric Acid (TBA) reaction [24]. For tissue and serum, 200 mg of brain tissue homogenized in PBS and 200 mL of serum were mixed with 2 mL of trichloroacetic acid (TCA) (20 %) and 2 mL of TBA (0.8 % in 0.25 N HCl), respectively, and incubated for 30 min at room temperature. 100 °C was heated. After centrifugation of the samples, the absorbance of the MDA-TBA complex was recorded at 532 nm, and the MDA concentration was calculated based on the standard formula and the molar extinction coefficient of 1.56 × 10^5^ M^−1^cm^−1^.

### 2.5 Bioinformatics analysis

Molecular docking was used to evaluate the effects of NAC, SMC, and rotenone on the antioxidant proteins Sod, Gpx, Cat, Nrf2, and Ho-1. Also, the effect of rotenone, which is known as an oxidative stress inducer and PD's model, on the structural characteristics of the aforementioned proteins was investigated. The 3D structures of the ligands were extracted from PubChem and converted to the appropriate PDBQT format for docking in PyRx with Open Babel. The crystallographic structures of the proteins were also obtained from the PDB database and processed with Discovery Studio software after removing water molecules and additional ligands. Then, using AutoDock Vina in the PyRx environment, docking was performed, and the active site of each enzyme was set as a Grid Box to examine the binding of ligands only in the functional site of the enzymes (Table 2). The binding energy was calculated, where more negative values correspond to stronger binding affinity. After docking, molecular interactions between ligands and amino acids of the active site of the enzymes, including hydrogen bonds, hydrophobic interactions, and van der Waals interactions, were analyzed with Discovery Studio. Finally, a 2D interaction map was drawn to visually display the ligand-protein interactions and provide more detailed information on how the compounds bind to the target enzymes.

**Table 2:** Coordinates of the Grid Box around the protein active site.

Protein/compound	Grid Center	Grid Center	Grid Center	Grid Size	Grid Size	Grid Size
Gpx/Rotenone	14.382	9.157	26.741	10.783	9.234	12.674
Gpx/NAC	13.905	8.763	25.318	10.459	9.874	11.980
Gpx/SMC	15.217	9.321	27.104	10.912	9.542	12.103
Sod/Rotenone	16.734	10.592	28.445	11.145	9.013	13.205
Sod/NAC	17.081	10.789	28.792	11.204	9.342	13.478
Sod/SMC	16.985	10.701	28.691	11.179	9.285	13.391
Cat/Rotenone	18.296	11.846	30.104	10.823	8.789	14.213
Cat/NAC	18.523	11.907	30.294	10.935	8.951	14.365
Cat/SMC	18.417	11.880	30.207	10.902	8.913	14.314
Nrf2/Rotenone	20.058	13.129	32.518	11.632	9.645	15.724
Nrf2/NAC	20.211	13.302	32.741	11.698	9.713	15.903
Nrf2/SMC	20.165	13.215	32.664	11.675	9.682	15.834
Ho-1/Rotenone	22.347	14.417	35.123	12.287	10.031	16.942
Ho-1/NAC	22.513	14.602	35.412	12.403	10.184	17.109
Ho-1/SMC	22.458	14.498	35.306	12.362	10.123	17.047

Cat: Catalase, Gpx: Glutathione peroxidase, Ho-1: Hemeoxygenase-1, NAC: N-Acetylcysteine, SMC: S-Methylcysteine, Sod: Superoxide dismutase.

The lncRNAs under investigation were selected due to their well-defined regulatory functions in cellular stress responses. Correspondingly, the gene targets included a master transcription factor in antioxidant defense, and its downstream effectors: *Ho1*, *Sod*, *Gpx*, and *Cat*. Interaction networks were constructed using Cytoscape. The input data consisted of two files: a node list defining molecular identities and functional types (e.g., enzyme, transcription factor, lncRNA), and an edge list indicating the type of biological interaction (e.g., transcriptional regulation, enzymatic cooperation, lncRNA interaction). The data sources were curated from public databases and literature, focusing on validated and predicted interactions localized in the cytoplasm. The network included both canonical pathways and inferred lncRNA-gene interactions.

### 2.6 Statistical analysis

The data from enzyme activity and gene expression were reported as mean ± standard deviation. For statistical comparison between groups, one-way analysis of variance (ANOVA) and Tukey's post hoc test were used for significant differences. Statistical analysis of data was performed with SPSS software version 20 (SPSS Inc., IBM Corp, Armonk, NY, USA), and *P* < 0.05 was considered as the level of statistical significance.

## 3 Results

### 3.1 Gene expression outcomes

**Fig. 2:**
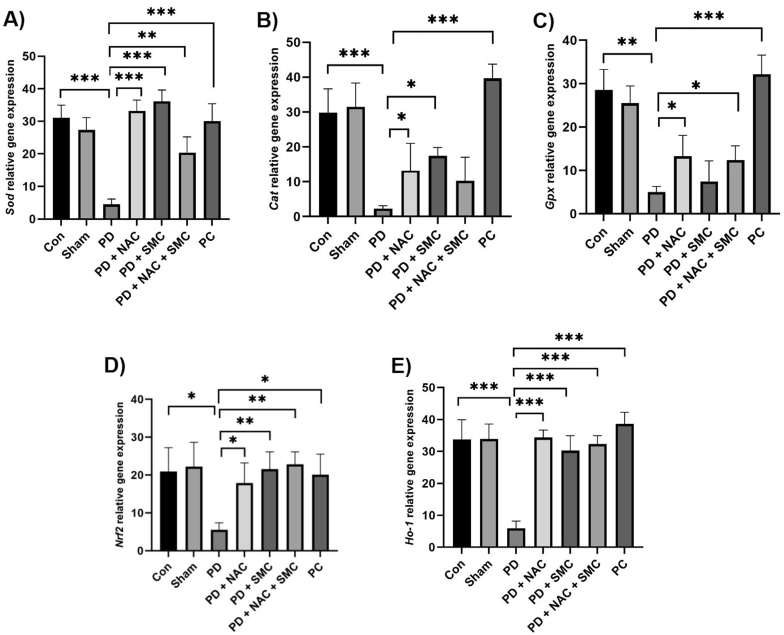
Results of the gene expression of *Sod*, *Cat*, *Gpx*, *Nrf2* and *Ho-1* in the brain tissue after treatment of mice with rotenone, NAC and SMC. Representation of changes in the activities of *Sod* (A), *Cat* (B), *Gpx* (C), *Nrf2* (D) and *Ho-1* (E) genes after treatments. The symbols ∗, ∗∗ and ∗∗∗ indicate significant differences less than 0.05, 0.01, and 0.001, respectively (n = 8). Con: Control Group, PD: Parkinson's disease, PD + NAC: PD group receiving NAC, PD + SM: PD group receiving SMC, PD + NAC + SMC: PD group receiving NAC and SMC, and PC: Positive control group receiving Levodopa.

Data analysis showed that there was a significant decrease in *Sod* gene expression in the PD group compared to the control group (*P* = 0.0003). Examination of the effect of NAC showed that this compound was able to significantly increase the level of Sod gene expression compared to the PD group (*P* < 0.001). Similarly, SMC also showed a positive effect on increasing the expression of this gene (*P* < 0.001). In addition, co-administration of NAC and SMC showed that *Sod* expression significantly increased compared to the PD group (*P* = 0.0048) (Fig. 2-A). Like *Sod*, *Cat* gene expression was also significantly decreased in the PD group compared to the control group (*P* < 0.001). The conducted studies showed that NAC (0.037) and SMC (0.046) administration in the PD group caused a significant increase in the expression of this gene compared to the untreated PD group. Also, the comparison between the PD's group and the positive control group showed that there was a significant difference in *Cat* expression between these two groups (*P* = 0.0004), which results indicate a possible protective effect of the studied compounds on oxidative stress associated with PDs (Fig. 2-B). In the study of *Gpx* expression, it was found that the expression level of this gene was significantly reduced in the PD group (*P* = 0.002). Meanwhile, NAC administration significantly increased the expression of *Gpx* (*P* = 0.023), but SMC had no significant effect on the expression level of this gene compared to the untreated PD group. However, the simultaneous study of the effect of NAC and SMC showed that the combination of these two substances significantly increased the expression of *Gpx* compared to the PD group (*P* = 0.017) (Fig. 2-C).

The results of the expression of *Nrf2* and *Ho-1* genes are shown in Fig. 2-D and Fig. 2-E, respectively. In addition, the analysis of the expression level of the *Nrf2* gene also showed that this regulatory factor was significantly reduced in the PD group compared to the control group (*P* = 0.038). Further investigation showed that both NAC and SMC increased the expression of this gene compared to the PD group (*P* = 0.019 and *P* = 0.004, respectively). Interestingly, co-administration of these two compounds further enhanced the expression of *Nrf2*, which was statistically significant (*P* = 0.002) (Fig. 2-D). These findings indicate that *Nrf2*, as a key factor in regulating antioxidant responses, is affected by these compounds and may play a role in improving PD's conditions. Examination of the expression of the *Ho-1* gene, which is one of the genes dependent on the *Nrf2* pathway, showed that it was significantly reduced in the PD group compared to the control group (*P* < 0.001). Administration of NAC and SMC separately significantly increased the expression level of *Ho-1* compared to the PD group (*P* < 0.001 and *P* < 0.001). Also, the combination of these two substances increased the expression of Ho-1 compared to the PD group (*P* < 0.001), indicating the potential role of these compounds in regulating cellular protective pathways (Fig. 2-E). Overall, the results of this study show that NAC and SMC compounds have a significant effect on increasing the expression of antioxidant genes and oxidative stress regulatory factors in PD. The increase in the expression of *Sod, Cat, Gpx, Nrf2,* and *Ho-1* genes due to the consumption of these compounds indicates their therapeutic potential as adjunctive approaches for managing oxidative stress linked to PD.

### 3.2 Examination of lncRNAs expression

**Fig. 3:**
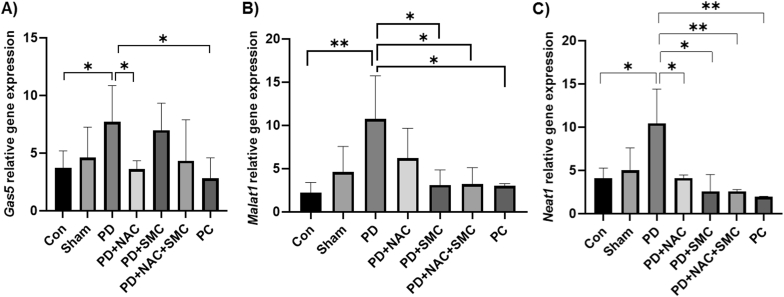
Results of the gene expression of lncRNAs in the brain tissue after treatment of mice with rotenone, NAC and SMC. Representation of changes in the activities of *Gas5* (A), *Malat1* (B) and *Neat1* (C) lncRNAs after treatments. The symbols ∗, ∗∗ and ∗∗∗ indicate significant differences less than 0.05, 0.01, and 0.001, respectively (n = 8). Con: Control group, PD: Parkinson's disease, PD + NAC: PD group receiving NAC, PD + SMC: PD group receiving SMC, PD + NAC + SMC: PD group receiving NAC and SMC, and PC: Positive control group receiving Levodopa.

In this study, the expression levels of the lncRNAs *Malat1*, *Neat1*, and *Gas5* were evaluated across different experimental groups (Fig. 3). *Gas5* expression was significantly increased in the PD group versus the healthy controls (*P* = 0.048), and NAC treatment markedly lowered its expression (*P* = 0.013). A significant difference in *Gas5* levels between the PD group and the positive control was also detected (*P* = 0.027) (Fig. 3-A). The results revealed a significant upregulation of *Malat1* in the Parkinson's disease (PD) group compared to the healthy control group (*P* = 0.002). Treatment with SMC significantly reduced Malat1 expression in comparison to the PD group (*P* = 0.033), and a combination of NAC and SMC led to an even further reduction (*P* = 0.019). A statistically significant difference in *Malat1* expression was also observed between the PD group and the positive control (*P* = 0.014) (Fig. 3-B). Similarly, *Neat1* expression was significantly elevated in the PD group compared to healthy controls (*P* = 0.044). Treatment with NAC led to a notable decrease in *Neat1* expression (*P* = 0.036), mirrored by SMC treatment (*P* = 0.029). Interestingly, co-treatment with NAC and SMC had a synergistic effect, producing a highly significant reduction compared to the PD group (*P* = 0.004), with a significant difference. Also, a significant difference was found between the PD group and the positive control (*P* = 0.003) (Fig. 3-C).

### 3.3 Results of enzyme activity and oxidative stress indices

**Fig. 4:**
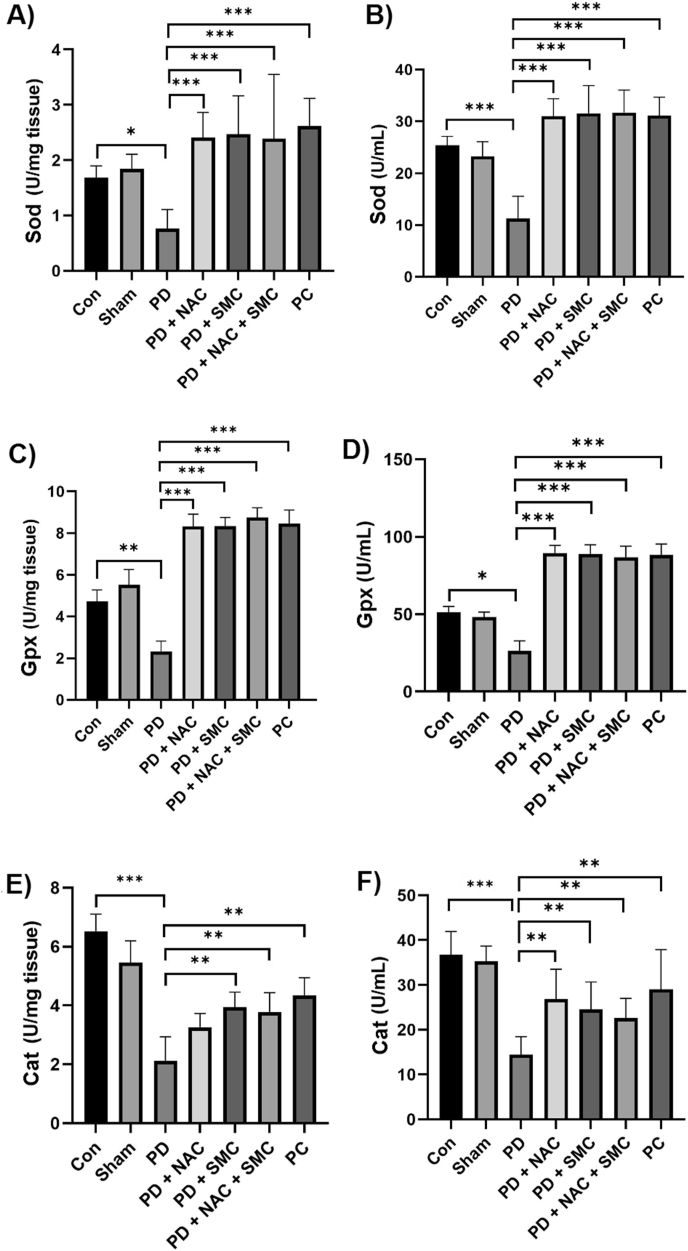
Results of the activities of Sod, Gpx, and Cat in serum and brain tissue. Representation of changes in the activities of Sod in brain tissue (A), Sod in serum (B), Gpx in brain tissue (C), Gpx in serum (D), Cat in brain tissue (E), and Cat in serum (F) enzymes after treatments. The symbols ∗, ∗∗ and ∗∗∗ indicate significant differences less than 0.05, 0.01, and 0.001, respectively (n = 8). Con: Control Group, PD: Parkinson's disease, PD + NAC: PD group receiving NAC, PD + SMC: PD group receiving SMC, PD + NAC + SMC: PD group receiving NAC + SMC, and PC: Positive control group receiving Levodopa.

Measurement results of antioxidant enzyme activity are shown in Fig. 4. The Sod enzyme activity in the serum and brain tissue of mice in the control group was 1.70 ± 25.43 and 1.69 ± 0.21 units/ml, respectively, while this level was significantly reduced in the PD group (4.25 ± 11.29 in serum and 0.35 ± 0.76 in brain tissue; *P* = 0.029 in tissue and *P* < 0.001 in serum), indicating a severe increase in oxidative stress caused by disease induction. NAC administration resulted in an increase in Sod enzymatic activity to 31.00 ± 3.37 in serum and 2.41 ± 0.45 in brain tissue compared to the PD group (*P* < 0.001). SMC treatment also resulted in a significant improvement, and the mean Sod activity increased to 31.57 ± 5.36 in serum and 2.47 ± 0.69in brain tissue (*P* < 0.001). In the NAC + SMC group, Sod activity was also significantly higher than the PD group, with a mean of 31.71 ± 4.35 in serum and 2.39 ± 1.17in brain tissue (*P* < 0.001) (Fig. 4-A and Fig. 4-B).

The Gpx enzyme activity in the serum and brain tissue of mice in the control group was 3.73 ± 51.35 and 4.74 ± 0.54 units/ml, respectively, while this level was significantly reduced in the PD group (26.30 ± 6.51 in serum and 2.32 ± 0.50 in brain tissue; *P* = 0.038 in serum and *P* = 0.004 in tissue), indicating an increase in oxidative stress caused by disease induction. NAC administration increased Gpx activity to 89.54 ± 4.86in serum and 8.32 ± 0.60in brain tissue compared to the PD group (*P* < 0.001). Treatment with SMC also resulted in a notable improvement, with the mean Gpx activity increasing to 89.12 ± 5.73 in serum and 8.35 ± 0.41 in brain tissue (*P* < 0.001). In the NAC + SMC group, Gpx activity was also significantly higher than that in the PD group, with a mean of 86.86 ± 7.10 in serum and 3.18 ± 0.46 in brain tissue (*P* < 0.001) (Fig. 4-C and Fig. 4-D).

Cat enzyme activity in serum and brain tissue of mice in the control group was 36.78 ± 5.15 and 6.52 ± 0.58 units/ml, respectively, while this level was significantly reduced in the PD group (14.48 ± 4.019 in serum and 2.12 ± 0.81 in brain tissue; *P* < 0.001), indicating an increase in the level of oxidative stress caused by disease induction. NAC administration increased Cat activity to 34.56 ± 6.15 in serum (*P* = 0.002), but there were no significant changes in brain tissue. Treatment with SMC markedly enhanced the enzyme activity, and the Cat activity level increased to 36.87 ± 6.67 in serum and 3.54 ± 0.52 in brain tissue compared to the PD group (*P* = 0.003 in serum and *P* = 0.009 in tissue). In the NAC + SMC group, the Cat activity was also significantly higher than the PD group with an average of 32.60 ± 4.42 in serum and 2.49 ± 0.66 in brain tissue (*P* = 0.008 in serum and *P* = 0.005 in tissue; Fig. 4-E and Fig. 4-F).

The results of the MDA and TAC tests are shown in Table 3. The results showed that the MDA level in the PD group was significantly reduced compared to the control group, both in serum (*P* < 0.001) and in brain tissue (*P* < 0.01), indicating increased lipid peroxidation and oxidative stress. In contrast, treatment with NAC, SMC, and the combination of NAC + SMC resulted in a significant increase in MDA in both serum and tissue compared to the PD group (*P* < 0.01). Also, the TAC level in the PD group was significantly reduced compared to the control group (*P* < 0.001), while treatment with NAC, SMC, and NAC + SMC resulted in a significant increase in this index in serum and brain tissue compared to the PD group (*P* < 0.01).

**Table 3:** MDA and TAC levels in serum and brain tissue in different groups.

Sample type	Groups	MDA (nmol/mL)	TAC (μmol/L)
**Serum**	Control	43.22 ± 1.20	329.98 ± 7.92
	PD	20.03 ± 1.20 ^∗∗∗^	143.34 ± 0.89 ^∗∗∗^
	NAC	57.25 ± 1.99^##^	400.18 ± 5.37^##^
	SMC	56.05 ± 1.91^##^	396.96 ± 5.10^##^
	NAC + SMC	55.06 ± 2.12^##^	406.07 ± 1.69^##^
	PC	55.43 ± 2.34^##^	405.18 ± 2.51^##^
**Brain Tissue**	Control	9.81 ± 0.66	318.54 ± 22.57
	PD	5.77 ± 3.39 ^∗∗^	143.65 ± 2.25 ^∗∗∗^
	NAC	12.88 ± 1.13^##^	408.29 ± 9.64^##^
	SMC	12.72 ± 1.36^##^	405.29 ± 3.50^##^
	NAC + SMC	12.72 ± 1.41^##^	402.74 ± 7.05^##^
	PC	10.77 ± 1.34^##^	406.74 ± 11.66^##^

Data are reported as mean ± standard deviation from 8 male mice per group. Tukey's test was used to compare between groups. Values with significant differences from the control group are indicated by ∗∗ (*P* < 0.01) and ∗∗∗ (*P* < 0.001). Also, comparison with the PD group is indicated by ^##^ (*P* < 0.01). MDA: Malondialdehyde, NAC: N-Acetylcysteine, PD: Parkinson's disease, SMC: S-Methylcysteine, TAC: Total antioxidant capacity.

### 3.4 Bioinformatics assay

**Fig. 5:**
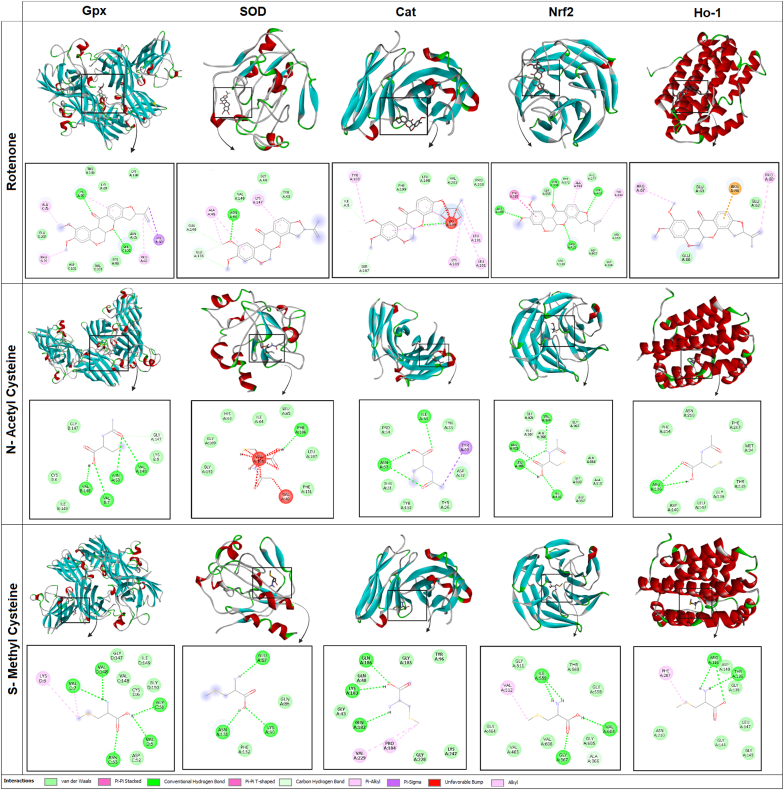
The figure illustrates the molecular interactions of the ligands rotenone, SMC, and NAC with five target proteins, *Gpx*, Sod, Cat, Nrf2 and Ho-1. The interactions between ligands and proteins are visualized using both 3D and 2D representations. The 2D interaction maps highlight key amino acid residues involved in binding. The explanation of the interactions is provided below the figure.

The results of molecular docking analysis of rotenone, NAC, and SMC with Sod, Gpx, Cat, Nrf2, and Ho-1 proteins are shown in Fig. 5. Data analysis showed that these compounds interact with key amino acids in the active site of the enzymes through hydrogen bonds and hydrophobic interactions. The SMC compound showed the highest binding affinity (−6.9 to −7.3 kcal/mol), indicating a stronger interaction and greater stability, while NAC had a moderate affinity (−6.8 to −7.2 kcal/mol), and rotenone showed the weakest binding (−6.5 to −6.9 kcal/mol). The Cat enzyme showed the highest binding affinity for SMC (−7.3 kcal/mol), while Ho-1 had the weakest interaction with rotenone (−6.5 kcal/mol). RMSD analysis showed that NAC and SMC had more stable interactions (RMSD lower: 2.1–2.7 Å, upper: 4.4–5.3 Å), while rotenone showed more fluctuations (RMSD lower: 2.7–3.2 Å, upper: 5.2–6.1 Å), suggesting increased structural flexibility within the binding site. In terms of molecular interactions, rotenone engages with critical residues including such as Asn46 in Sod, Ser102 in Gpx, Lys189 in Cat, and Arg415 in Nrf2 in enzymes through hydrogen bonds and π-π stacking, while NAC is mainly stabilized through hydrogen bonds with Val146 in Gpx, Phe186 in Sod, Ile34 in Nrf2, and Arg136 in Ho-1 and hydrophobic interactions. The SMC compound also showed the highest stability by forming hydrogen and hydrophobic bonds with Val146 in Gpx, Asn131 in Sod, Gln186 in Cat, and Gly367 in Nrf2. These results indicate that SMC, as the most stable ligand, has a higher potential to regulate the activity of antioxidant enzymes and can have a greater impact on the structural stability and function of these proteins; it also shows a significant advantage over rotenone in competitive binding with enzymes and other molecules under study.

**Fig. 6:**
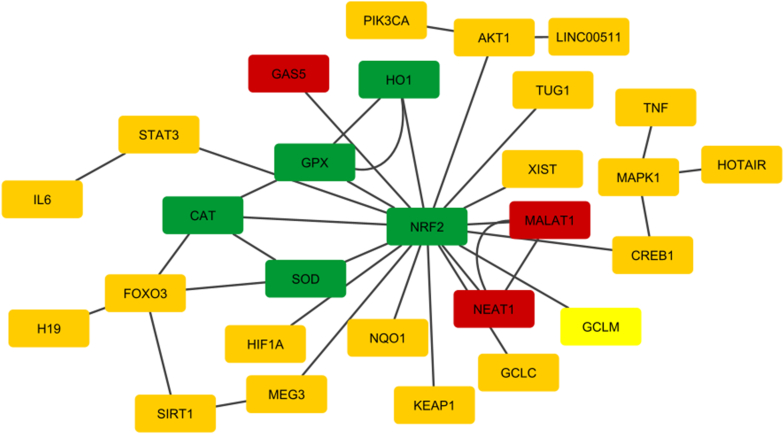
Gene interaction network deduced from Cytoscape. Interaction network showing *Nrf2* as a central regulator of antioxidant genes *Ho1*, *Sod*, *Cat*, and *Gpx* (green). lncRNAs *Neat1*, *Malat1*, and *Gas5* (red) interact with *Nrf2* and other molecules involved in the network (orange), highlighting their potential modulatory roles.

The interaction network (Fig. 6) showed that *Nrf2* plays a key role in controlling the body's defense against oxidative stress. It directly affects the expression of important antioxidant enzymes like *Ho-1*, *Sod*, *Cat*, and *Gpx*. These connections were identified as transcriptional regulation. Further analysis showed that *Sod* and *Cat* work together in a sequence to help reduce oxidative damage. When lncRNAs were added to the network, new layers of regulation became clear. *Neat1* and *Malat1* were linked to *Nrf2*, indicating they may contribute to regulating its activity or stabilizing its mRNA in the cytoplasm. *Gas5*, known for blocking miRNAs, also showed links to antioxidant enzymes, possibly by acting as a sponge or through RNA interactions. The network highlights a coordinated response to oxidative stress not just in the nucleus, but also in the cytoplasm. It suggests that lncRNAs may help regulate the *Nrf2* pathway after transcription, adding more complexity to how cells protect themselves.

## 4 Discussion

Parkinson's disease, as one of the most common neurodegenerative disorders, is characterized by the progressive destruction of dopaminergic neurons in the substantia nigra of the brain, and oxidative stress plays a key role in its pathophysiology [3]. This study investigated the protective effects of NAC and SMC against oxidative stress and their influence on antioxidant gene expression and enzyme activity in a rotenone-induced Parkinson's disease model. Rotenone administration significantly reduced the expression of *Sod*, *Gpx*, *Cat*, *Nrf2*, and *Ho-1* genes, along with a decline in related enzyme activities and total antioxidant capacity, indicating severe oxidative stress. Treatment with NAC and SMC, alone or combined, markedly upregulated these genes, restored enzyme activities, increased TAC, and decreased lipid peroxidation, demonstrating enhanced antioxidant defense. These findings suggest that NAC and SMC protect neurons from oxidative damage, partly through activation of Nrf2 and reinforcement of antioxidant pathways. It has been reported that NAC protects neurons against oxidative damage by increasing glutathione levels and activating Nrf2 [25]. SMC is also known as a garlic-derived compound with the ability to inhibit ROS production and increase the activity of antioxidant enzymes [17]. The molecular mechanism proposed in the present study involves the release of NAC and SMC into the cellular environment, inhibition of ROS, stimulation of Nrf2 translocation to the nucleus, and activation of ARE-controlled antioxidant gene expression [26]. This pathway, especially in neurons under oxidative stress, leads to inhibition of cell death and improvement of neuronal survival by reducing the activity of Nrf2-degrading kinases and increasing Ho-1 expression. Therefore, NAC and SMC can be considered as appropriate options for therapeutic interventions in neurodegenerative diseases, including Parkinson's, as modulators of antioxidant defense pathways [27]. In agreement with our results, several recent studies have demonstrated that naturally derived antioxidant compounds exert significant protective effects against oxidative stress through activation of the Nrf2/Ho-1 signaling cascade. These compounds were shown to restore the activities of key antioxidant enzymes and suppress lipid peroxidation, thereby maintaining cellular redox equilibrium. Such findings further highlight the pivotal contribution of Nrf2-mediated transcriptional regulation in sustaining antioxidant defense under oxidative stress conditions [[28], [29], [30], [31], [32], [33]].

Furthermore, the expression profiles of the lncRNAs *Malat1*, *Neat1*, and *Gas5* were significantly dysregulated in the Parkinson's disease model, with *Malat1* and *Neat1* showing notable upregulation, which has been associated with enhanced oxidative stress and neuroinflammation. In contrast, *Gas5*, a lncRNA known to influence cell survival and antioxidant pathways, also exhibited altered expression. Treatment with NAC and SMC, either alone or in combination, led to a significant normalization of these lncRNAs' expression levels. This suggests that the protective effects of these compounds may extend beyond classical antioxidant gene induction, potentially acting through modulation of lncRNA-miRNA-NRF2 regulatory axes involved in oxidative stress and neuronal survival. *Malat1* suppresses *Nrf2* through epigenetic mechanisms, leading to inflammasome activation and increased reactive oxygen species generation in both PD mouse and microglial cell models, thereby contributing to oxidative stress and neuroinflammatory responses [11]. In addition, increased expression of *Neat1* inhibits the *Nrf2* signaling pathway, thereby promoting neuronal injury and cognitive deficits, providing a critical reference for understanding the underlying molecular mechanisms of neuronal damage and guiding potential therapeutic strategies [14]. It has been demonstrated that Ginsenoside Rg1 reduced oxidative stress in corticosterone-stimulated PC12 cells by increasing *Nrf2* and *Ho*-1 expression while downregulating *Gas5*. Silencing *Gas5* confirmed its inhibitory effect on *Nrf2* via EZH2. Similar effects were observed in chronically stressed mice, highlighting that Rg1's protective antioxidant action is mediated through GAS5 [34,35].

In the bioinformatics analysis, the results showed that NAC compounds and especially SMC can effectively interact with key antioxidant enzymes and factors such as Sod, Gpx, Cat, Nrf2, and Ho-1. Molecular docking data indicated that SMC, with the lowest binding energy and RMSD values compared to other ligands, established the most stable and strong binding. This binding was mainly formed through hydrogen bonds with key residues such as GLY131, ASN146, and GLN186, as well as hydrophobic interactions with ILE149 and VAL312, which indicates the stabilization of the ligand position in the active site of the protein. NAC also showed stabilizing interactions, albeit moderately. In contrast, rotenone, with its higher volatility and weaker binding energy, did not produce stabilizing interactions. These findings are also consistent with previous studies. For example, through a computational model of recombinant human α-glucosidase with NAC, it was demonstrated that NAC enhances the structural stability of the enzyme [36]. Based on the proposed mechanism, NAC and SMC probably induce the transcription of antioxidant genes by stabilizing Nrf2 protein and facilitating its translocation to the nucleus. Additionally, direct interaction of these compounds with antioxidant enzymes may enhance their structural stability and improve their catalytic function. These proposed molecular pathways not only justify the bioinformatics findings but also are consistent with the in vivo experimental results, and collectively strengthen the potential therapeutic role of these compounds in modulating oxidative stress and neuroprotection in the Parkinson's model.

With the accelerating discovery of links between long noncoding RNAs and various human disorders, this area of research has emerged as a promising “goldmine” for uncovering new molecular targets for disease diagnosis and therapy [[37], [38], [39], [40]]. Multiple studies indicate that lncRNAs are abundant in the central nervous system (CNS) and have a key role in brain function as well as many neurological disorders, especially in CNS injuries [41]. Although numerous lncRNA targets have been identified, most still require experimental validation, and their clinical translation remains in early stages. lncRNAs show significant potential as molecular targets in CNS injuries, as their diverse regulatory roles and widespread dysregulation in neurological disorders highlight their importance in disease mechanisms. Targeting lncRNAs could thus provide innovative and precise diagnostic and therapeutic strategies that may surpass existing approaches. Moreover, since technologies designed for other RNA molecules (such as mRNAs and miRNAs) can be adapted for lncRNA research, the development of lncRNA-based clinical tools is expected to progress rapidly and cost-effectively [42].

Neurodegenerative disorders such as Parkinson's disease are closely associated with oxidative stress and dysregulated gene expression in neural cells. Among emerging molecular regulators, long non-coding RNAs have gained attention as key modulators of neuronal survival, oxidative balance, and neuroinflammatory signaling [43]. Mounting evidence suggests that aberrant lncRNA expression contributes to the progression of Parkinson's disease by influencing mitochondrial function, apoptosis, and redox homeostasis [44]. Meanwhile, phytochemicals, particularly those derived from traditional medicinal plants, exhibit remarkable neuroprotective potential through their antioxidant and anti-inflammatory properties. Recent studies indicate that certain phytochemicals can modulate lncRNA expression, thereby restoring normal cellular signaling and mitigating oxidative injury in dopaminergic neurons. By targeting oxidative stress–related pathways and lncRNA-mediated gene networks, phytochemicals offer a promising avenue for precision therapy in Parkinson's disease, integrating molecular regulation with natural neuroprotection [45].

This study has several limitations that should be acknowledged. First, although the expression levels of specific lncRNAs were assessed, their regulatory relationship with Nrf2 was inferred only through bioinformatics predictions and not experimentally confirmed by techniques such as RNA immunoprecipitation. Second, the analysis was limited to gene expression and biochemical parameters without validation at the protein level, which could provide a more comprehensive mechanistic understanding. Furthermore, the study did not include behavioral assessments, which restricts the translational relevance of the findings to clinical manifestations of Parkinson's disease. Finally, the exclusive use of male mice, the relatively short duration of exposure, and the fixed dosing regimen may limit the generalizability of the outcomes. Future studies are therefore encouraged to integrate molecular, protein-level, and behavioral evaluations to elucidate the precise mechanistic pathways and enhance the translational potential of these findings.

## 5 Conclusion

The findings of this research suggest that NAC and SMC compounds play an effective role in boosting antioxidant defense mechanisms and attenuating lipid peroxidation in Parkinson's by activating the *Nrf2*/*Ho-1* signaling pathway and increasing the expression and activity of antioxidant enzymes, including *Sod*, *Gpx*, and *Cat*. The increase in *Nrf2* and *Ho-1* levels in the brain, along with the decrease in oxidative stress indicators such as MDA and the increase in TAC, reflects the upregulation of cellular protective responses against oxidative damage. Overall, the results of the study emphasize the potential capacity of these natural compounds in strengthening the antioxidant system and supporting adjunctive treatment strategies in the treatment of PD.

## CRediT authorship contribution statement

Sahar Yaqubi: Data curation, Formal analysis. Writing – original draft. Bagher Seyedalipour: Conceptualization, Supervision, Writing – original draft. Mohammad Karimian: Conceptualization, Writing – original draft.

## Ethical approval

All stages of the experiment were carried out according to the guidelines of the Bioethics Committee of the University of Mazandaran (Bioethics Code: IR.UMZ.REC.1403.092).

## Founding information

This study was conducted with personal funding.

## Declaration of competing interest

The authors declare that they have no known competing financial interests or personal relationships that could have appeared to influence the work reported in this paper.

## Data Availability

The data generated during the current project are available from the corresponding author on reasonable request.

## Abbreviation:

ANOVA: One-way analysis of variance, AREs: Antioxidant response elements, Cat: Catalase, Gpx: Glutathione peroxidase, Ho-1: Hemeoxygenase-1, lncRNAs: long non-coding RNAs, MDA: Malondialdehyde, NAC: N-Acetyl Cysteine, Nrf2: Nuclear factor erythroid 2-related factor 2, PD: Parkinson's disease, ROS: Reactive oxygen species, SMC: S-Methyl Cysteine, Sod: Superoxide dismutase, TAC: Total antioxidant capacity, TCA: Trichloroacetic acid.
